# Anti-Amnesia-like Effect of *Pinus densiflora* Extract by Improving Apoptosis and Neuroinflammation on Trimethyltin-Induced ICR Mice

**DOI:** 10.3390/ijms241814084

**Published:** 2023-09-14

**Authors:** Min Ji Go, Jong Min Kim, Hyo Lim Lee, Tae Yoon Kim, Seung Gyum Joo, Ju Hui Kim, Han Su Lee, Dae-Ok Kim, Ho Jin Heo

**Affiliations:** 1 Division of Applied Life Science (BK21), Institute of Agriculture and Life Science, Gyeongsang National University, Jinju 52828, Republic of Korea; alswl9245@gnu.ac.kr (M.J.G.); myrock201@gnu.ac.kr (J.M.K.); gyfla059@gnu.ac.kr (H.L.L.); kty8747@gnu.ac.kr (T.Y.K.); 2021210440@gnu.ac.kr (S.G.J.); zkfkapflove@nate.com (J.H.K.); ns3005@naver.com (H.S.L.); 2 Department of Food Science and Biotechnology, Kyung Hee University, Yongin 17104, Republic of Korea; dokim@khu.ac.kr

**Keywords:** Korean red pine (*Pinus densiflora*) bark, Alzheimer’s disease, cholinergic system, apoptosis, neuroinflammation, mitochondrial function

## Abstract

This study was conducted to investigate the anti-amnestic property of Korean red pine bark extract (KRPBE) on TMT-induced cognitive decline in ICR mice. As a result of looking at behavioral function, the consumption of KRPBE improved the spatial work ability, short-term learning, and memory ability by Y-maze, passive avoidance, and Morris water maze tests. KRPBE suppressed antioxidant system damage by assessing the SOD activity, reduced GSH content, and MDA levels in brain tissue. In addition, it had a protective effect on cholinergic and synaptic systems by regulating ACh levels, AChE activity, and protein expression levels of ChAT, AChE, SYP, and PSD-95. Also, the KRPBE ameliorated TMT-induced mitochondrial damage by regulating the ROS content, MMP, and ATP levels. Treatment with KRPBE suppressed Aβ accumulation and phosphorylation of tau and reduced the expression level of BAX/BCl-2 ratio and caspase 3, improving oxidative stress-induced apoptosis. Moreover, treatment with KRPBE improved cognitive dysfunction by regulating the neuro-inflammatory protein expression levels of p-JNK, p-Akt, p-IκB-α, COX-2, and IL-1β. Based on these results, the extract of Korean red pine bark, which is discarded as a byproduct of forestry, might be used as an eco-friendly material for functional foods or pharmaceuticals by having an anti-amnesia effect on cognitive impairment.

## 1. Introduction

Alzheimer’s disease (AD) is one of the most lethal brain disorders in the elderly population [1]. AD refers to a neurodegenerative disorder accompanied by memory deficits, cognitive ability decline, and behavioral changes [1]. The number of patients with AD, the most common type of dementia, is projected to exceed 130 million by 2050 [2]. AD is considered one of the diseases with high annual health care costs, which causes economic burden on individuals and society [2]. The cause of sporadic AD, which accounts for more than 95% of AD cases, include the amyloid and tau aggregation hypothesis, inflammatory hypothesis, cholinergic hypothesis, mitochondrial cascade hypothesis, neurovascular hypothesis, and lymphatic system hypothesis [3].

Oxidative stress induced amyloid beta (Aβ) deposition and hyperphosphorylation of tau promote various mechanisms such as reactive oxygen species (ROS) generation and increase synaptic and neuronal cell loss and is involved in the development of AD [4]. Free radicals that generate oxidative stress are very unstable and highly reactive due to unpaired electrons [5]. Therefore, free radicals generated in the body are toxic, and if not removed or neutralized, react with lipids, proteins, and nucleic acids to cause cell dysfunction and cell death [5]. In particular, the central nervous system (CNS) is vulnerable to free radical damage due to its high oxygen consumption rate, high lipid content, and relatively insufficient antioxidant enzymes compared to other tissues [6]. In addition, oxidative stress such as ROS generated during neurodegeneration induces lipid peroxidation and enhances the activity of acetylcholinesterase (AChE), resulting in an imbalance of the cholinergic system [7]. Oxidative stress can be triggered by an inflammatory reaction and Aβ and neurofibrillary tangles (NFTs) generated by oxidative stress induce or promote neuroinflammation by activating microglia and secreting tumor necrosis factor-α (TNF-α) [8,9,10]. Therefore, various approaches are reported to be more effective than a single targeted therapy in terms of the interaction between oxidative stress, Aβ and p-tau, and the complex pathology of AD. In particular, natural product mixtures and extracts are important for the prevention and treatment of multifactorial AD because they have various physiologically active compounds and neuroprotective mechanisms [11].

Trimethyltin (TMT) is an organotin compound that causes intense behavioral and cognitive impairment in both humans and laboratory animals [12,13]. It is a neurotoxic substance that acts specifically in the hippocampus and causes the loss of nerve cells and behavioral disorders due to neuroinflammation [13]. Furthermore, TMT-induced neurodegeneration shares pathological mechanisms with many neurodegenerative diseases, such as oxidative stress and apoptosis. Therefore, TMT has been a useful tool for inducing neuropathological models and was used as a neurobehavioral disorder inducer in this study [6].

Korean red pine (*Pinus densiflora*), one of the most common pine species native to Korea, has been used as a traditional medicine for many ailments [14]. Pine bark is considered a byproduct of the timber industry and is treated as waste with no value [15]. However, pine bark extract (PBE) not only contains oligomeric procyanidin, which is a strong antioxidant, but also contains various phenolic compounds such as catechin, protocatechuic acid, taxifolin, and vanillin [14,15]. In addition, PBE is reported to have antihypertensive, anti-inflammatory, antioxidant, anti-lipogenic, and hair growth promoting effects [14,15,16]. It has phenolic compounds (catechin and taxifolin) and flavonoids (procyanidin B1 and B2) similar to the composition of pycnogenol (maritime pine *Pinus pinaster* bark extract, PYC) [17]. However, studies reported on anti-amnestic effects of KRPBE in neurodegenerative models are insufficient. Thus, in this study, the mechanism of delaying and suppressing the effects of AD induced by multifactorial factors by treatment with KRPBE, a natural material, was investigated.

## 2. Results

### 2.1. Behavioral Tests

#### 2.1.1. Y-Maze Test

**Figure 1:**
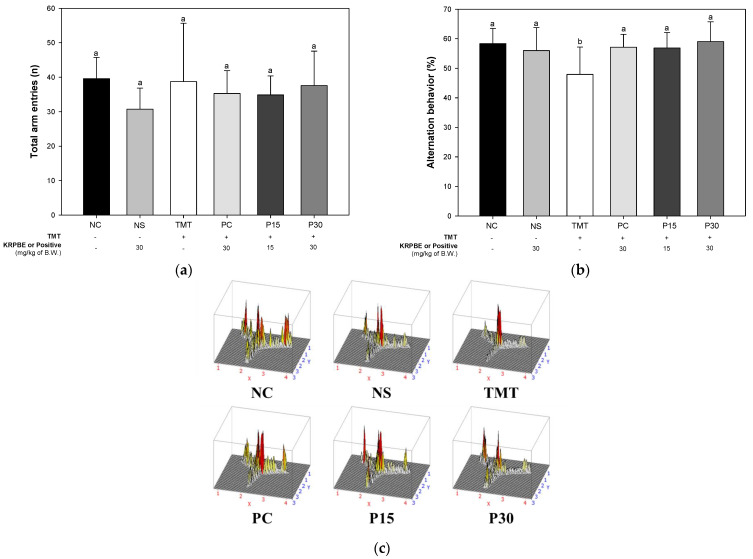
Protective effect of KRPBE on Y-maze test in TMT-induced cognitive impaired mice. (**a**) Number of arm entries in training session; (**b**) Alternation behavior in test session; (**c**) A 3D image of path tracking in the test session. The results shown are mean ± SD (*n* = 7). Data were statistically considered at *p* < 0.05 and different small letters represent statistical differences.

The Y-maze test was performed to evaluate the spatial perception ability in the form of short-term memory (Figure 1). The number of total arm entries was not significantly different between each group (Figure 1a). As the result of alternation behavior of the mice in the test session, there was no significant difference between the normal control (NC) and the normal sample (NS) groups (58.37% and 55.99%, respectively) (Figure 1b). The TMT group (47.91%) presented a decrease in the number of alternation behavior. However, the alternation behavior of the positive control (PC) group (57.12%) was significantly improved compared to the TMT group. Also, those of the P15 and P30 groups (56.85% and 59.04%, respectively) were significantly improved compared to alternation behavior of the TMT group. In addition, to observe the behavioral pattern of the mice during the test session, their movements were presented in Figure 1c.

#### 2.1.2. Passive Avoidance Test

**Figure 2:**
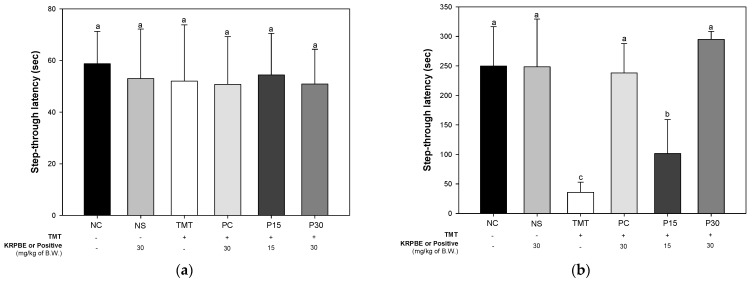
Protective effect of KRPBE on passive avoidance test in TMT-induced cognitive impaired mice. (**a**) Step-through latency time in the training session. (**b**) Step-through latency time in test session. The results shown are mean ± SD (*n* = 7). Data were statistically considered at *p* < 0.05 and different small letters represent statistical differences.

The passive avoidance test was performed to evaluate short-term learning and memory abilities (Figure 2). In the training session, there was no significant difference between each group in the latency (Figure 2a). After 24 h, the step-through latency time was not significantly different between the NC and NS groups (249.57 s and 248.43 s, respectively) in the test session (Figure 2b). The step-through latency time of the TMT group (35.86 s) was significantly reduced compared to the NC group. However, the PC group (238.00 s) was significantly increased compared to the step-through latency time of the TMT group. Also, the step-through latency time of the P15 and the P30 groups (101.43 s and 294.86 s, respectively) were significantly improved compared to TMT group.

#### 2.1.3. Morris Water Maze Test

**Figure 3:**
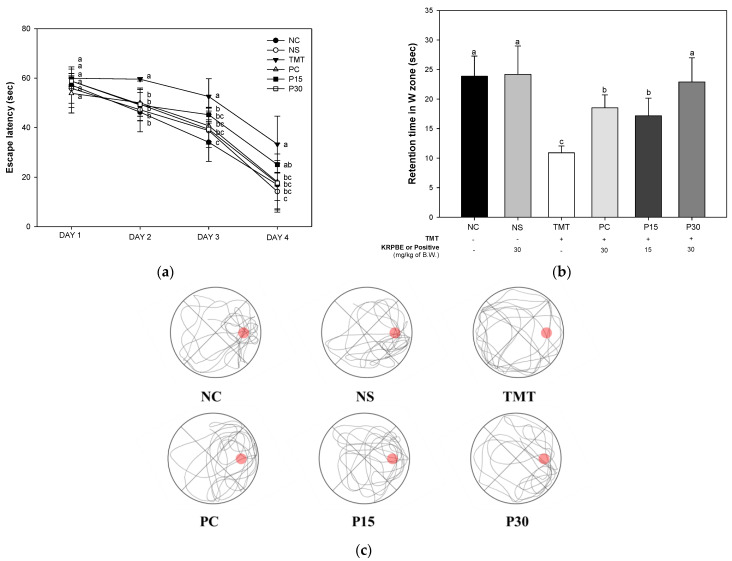
Protective effect of KRPBE on Morris water maze test in TMT-induced cognitive impaired mice. (**a**) Escape latency in the training session; (**b**) Retention time on W zone in the probe trail; (**c**) Swimming pattern visualization image tracked by video tracking system. The results shown are mean ± SD (*n* = 7). Data were statistically considered at *p* < 0.05 and different small letters represent statistical differences.

The Morris water maze test was conducted to evaluate spatial memory, long-term learning, and memory abilities (Figure 3). On the first day of training sessions, there was no significant difference between each group and it was confirmed that there was no difference in exercise capacity. On the fourth day of training sessions, the escape latency time was not significantly different between the NC and NS groups (16.95 s and 14.20 s, respectively) (Figure 3a). The escape latency time of the TMT group (33.31 s) was significantly delayed compared to the NC group. The PC group (18.07 s) was decreased compared to the escape latency time of the TMT group. The P15 group (25.09 s) did not show a significant difference from the TMT group. On the other hand, the P30 group (17.61 s) decreased significantly compared to the escape latency time of the TMT group. In the probe trial, the retention time in the W zone was not significantly different between the NC and NS groups (23.86 s and 24.18 s, respectively) (Figure 3b). The retention time of the TMT group (10.91 s) was significantly reduced compared to the NC group. The retention time of the PC group (18.54 s) was increased compared to the TMT group. In contrast, those P15 and P30 groups (17.18 s and 22.90 s, respectively) were significantly improved compared to the TMT group. Also, the result of observing the swimming pattern of the mice through the video tracking system during the probe trial is shown in Figure 3c.

### 2.2. Antioxidant System

**Figure 4:**
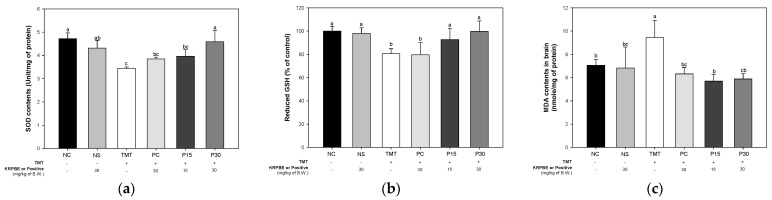
Effect of KRPBE on antioxidant system in TMT-induced cognitive impaired mice. (**a**) SOD contents; (**b**) Reduced GSH level; (**c**) MDA contents in brain tissue. The results shown are mean ± SD ((**a**), *n* = 5 and (**b**,**c**), *n* = 7). Data were statistically considered at *p* < 0.05 and different small letters represent statistical differences.

Antioxidant system evaluation was performed to confirm the effect of KRPBE on oxidative stress induced by TMT toxicity (Figure 4). As a result of measuring the contents of superoxide dismutase (SOD), the content of SOD was significantly different between the NC and NS groups (4.72 Unit/mg of protein and 4.31 Unit/mg of protein, respectively) (Figure 4a). The SOD content of the TMT group (3.44 Unit/mg of protein) showed a significant decrease compared to the NC group. The PC group (3.85 Unit/mg of protein) and P15 group (3.97 Unit/mg of protein) did not show a significant difference compared to the TMT group. On the other hand, the P30 group was (4.59 Unit/mg of protein) significantly increased compared to the TMT group.

As result of measuring the level of reduced glutathione (GSH), a representative antioxidant, the levels of the reduced GSH were not significantly different between the NC and NS groups (100% and 96.29% of control) (Figure 4b). The reduced GSH levels of TMT group (78.06%) were significantly reduced compared to the NC group. The PC group (79.60%) did not show a significant difference compared to the TMT group. On the other hand, those P15 and P30 groups (89.30% and 94.84%, respectively) were significantly improved compared to the reduced GSH levels of the TMT group.

Malondialdehyde (MDA) levels were measured to investigate free radical induced damage (Figure 4c). The content of MDA was not significantly different between the NC and NS groups (7.07 nmol/mg of protein and 6.83 nmol/mg of protein, respectively). The TMT group (9.45 nmol/mg of protein) increased significantly compared to the NC group. On the other hand, the PC group (6.32 nmol/mg of protein) was significantly reduced compared to the TMT group. Also, those P15 and P30 groups (5.70 nmol/mg of protein and 5.89 nmol/mg of protein, respectively) were significantly reduced compared to the MDA levels of the TMT group.

### 2.3. Cholinergic System and Synaptic Function

**Figure 5:**
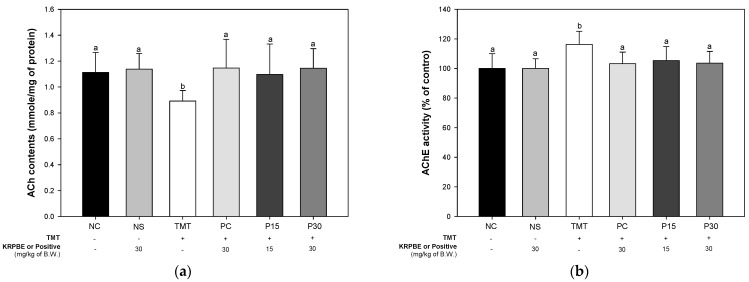
Effect of KRPBE on cholinergic system in TMT-induced cognitive impaired mice. (**a**) ACh contents; (**b**) AChE activity in brain tissue. The results shown are mean ± SD (*n* = 7). Data were statistically considered at *p* < 0.05 and different small letters represent statistical differences.

Acetylcholine (ACh) content and AChE activity were evaluated to confirm the effect of KRPBE on TMT-induced cholinergic system dysfunction (Figure 5). In the results of measuring the contents of neurotransmitter ACh, there was no significant difference between the NC and NS groups (1.11 mmol/mg of protein and 1.14 mmol/mg of protein, respectively) (Figure 5a). The ACh contents of the TMT group (0.89 mmol/mg of protein) were significantly reduced compared to NC group. On the other hand, the PC group (1.15 mmol/mg of protein) was significantly increased compared to the TMT group. Those P15 and P30 groups (1.10 mmol/mg of protein and 1.15 mmol/mg of protein, respectively) were significantly improved compared to the ACh contents of the TMT group.

As a result of measuring the activity of the AChE, there was no significant difference between the NC and NS groups (100% of control and 100.08% of control, respectively) (Figure 5b). The AChE activity of the TMT group (116.28% of control) was significantly reduced compared to the NC group. However, The PC group (103.28% of control) was reduced compared to the TMT group. Also, those P15 and P30 groups (105.32% of control and 103.64% of control, respectively) were significantly reduced compared to the AChE activity of the TMT group.

**Figure 6:**
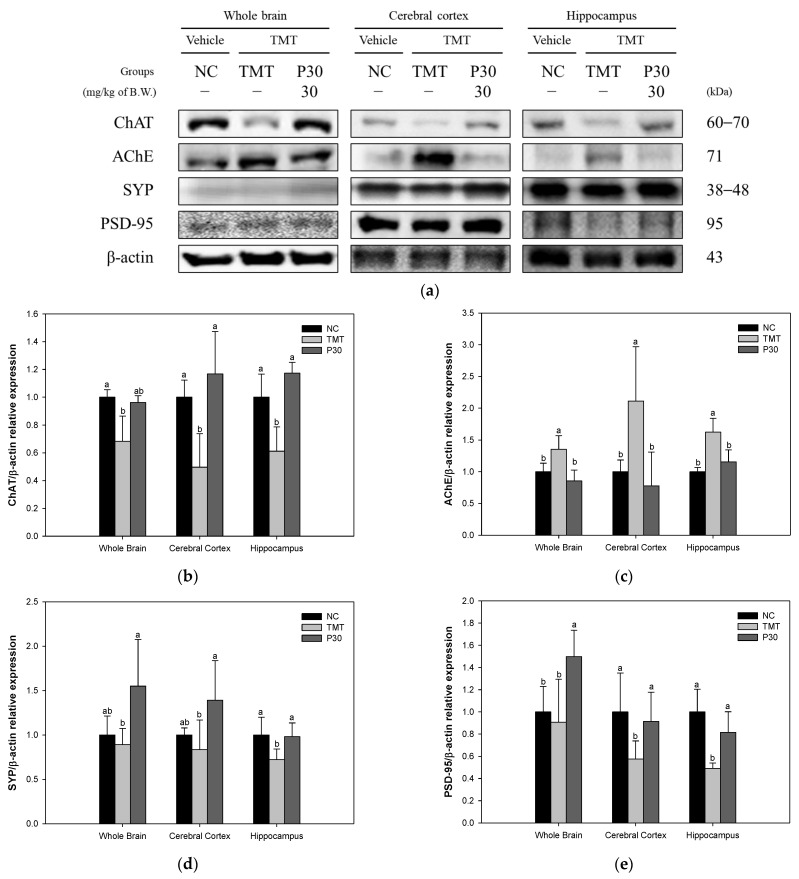
Ameliorating effect of KRPBE on cholinergic dysfunction and synaptic toxicity on the TMT-induced cognitive impaired mice. (**a**) Western blot band image: protein expression levels of (**b**) ChAT; (**c**) AChE; (**d**) SYP; and (**e**) PSD-95 in the whole brain, cerebral cortex, and hippocampus. The results shown are mean ± SD (*n* = 3). Data were statistically considered at *p* < 0.05 and different small letters represent statistical differences.

In addition, a Western blot was performed to investigate the protein expression level related to TMT-induced cholinergic dysfunction and synaptic toxicity (Figure 6). The expression level of choline acetyltransferase (ChAT), an enzyme that synthesizes acetyl-CoA and choline to produce ACh in the cholinergic system, was significantly decreased in the TMT group (68.35%, 49.71%, and 61.20%) compared to the NC group (Figure 6b). On the other hand, the P30 group (96.14%, 116.78%, and 117.30%, respectively) was significantly increased compared to the TMT group in cerebral cortex and hippocampus tissue. The AChE expression level of the TMT group (135.35%, 211.14%, and 162.45%) was significantly increased compared to the NC group (Figure 6c). On the other hand, the P30 group (85.49%, 77.82%, and 115.57%) was significantly decreased compared to the TMT group. Next, synaptophysin (SYP) and postsynaptic density protein-95 (PSD-95) were measured to investigate the improvement effect of KRPBE on TMT-induced synaptic toxicity (Figure 6d,e). The SYP expression level of the TMT group (89.13%, 83.55%, and 72.21%) was decreased compared to the NC group (Figure 6d). The P30 group (154.96%, 139.08%, and 98.10%) was significantly increased compared to the TMT group. In addition, the PSD-95 expression level of the TMT group (90.61%, 57.49%, and 49.06%) was decreased compared to the NC group (Figure 6e). However, the P30 group (149.82%, 91.36%, and 81.48%) was significantly increased compared to the TMT group.

### 2.4. Mitochondrial Function

**Figure 7:**
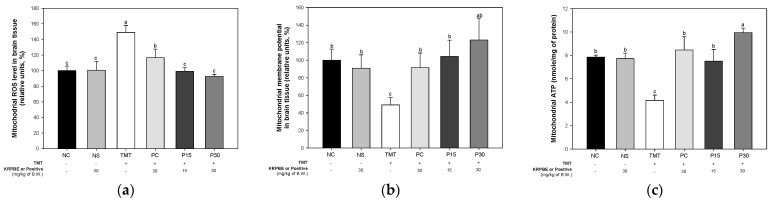
Ameliorating effect of KRPBE on mitochondrial dysfunction in TMT-induced cognitive impaired mice. (**a**) Mitochondrial ROS contents; (**b**) MMP level; (**c**) Mitochondrial ATP level in brain tissue. The results shown are mean ± SD (*n* = 5). Data were statistically considered at *p* < 0.05 and different small letters represent statistical differences.

ROS content, mitochondrial membrane potential (MMP), and adenosine triphosphate (ATP) levels were evaluated to investigate the improvement effect of KRPBE on mitochondrial dysfunction (Figure 7). The mitochondrial ROS content was not significantly different between the NC and NS groups (100% and 100.54% of control, respectively) (Figure 7a). The TMT group (148.97% of control) was significantly increased compared to the NC group. On the other hand, the mitochondrial ROS content of the PC group (116.61%) was increased compared to the TMT group. In addition, the P15 and P30 groups (99.10% of control and 92.59% of control, respectively) were significantly improved compared to the TMT group. As a result of examining the MMP level, there was no significant difference between the NC and NS groups (100% and 90.97%, respectively) (Figure 7b). The MMP level of the TMT group (49.09%) presented was significantly reduced compared to the NC group. However, the PC group (91.61%) was significantly increased compared to the MMP level of the TMT group. Those P15 and P30 groups (104.47% and 123.10%, respectively) were significantly improved compared to the TMT group. In the ATP level, there was no significant difference between the NC and NS groups (7.85 nmol/mg of protein and 7.73 nmol/mg of protein, respectively) (Figure 7c). The ATP levels of the TMT group (4.16 nmol/mg of protein) were significantly decreased compared to the NC and NS groups. On the other hand, the PC group (8.46 nmol/mg of protein) was increased compared to the TMT group. Also, the P15 and P30 groups (7.51 nmol/mg of protein and 9.94 nmol/mg of protein, respectively) were significantly improved compared to the mitochondrial ATP levels of the TMT group.

### 2.5. Aβ and Tau Contents

**Figure 8:**
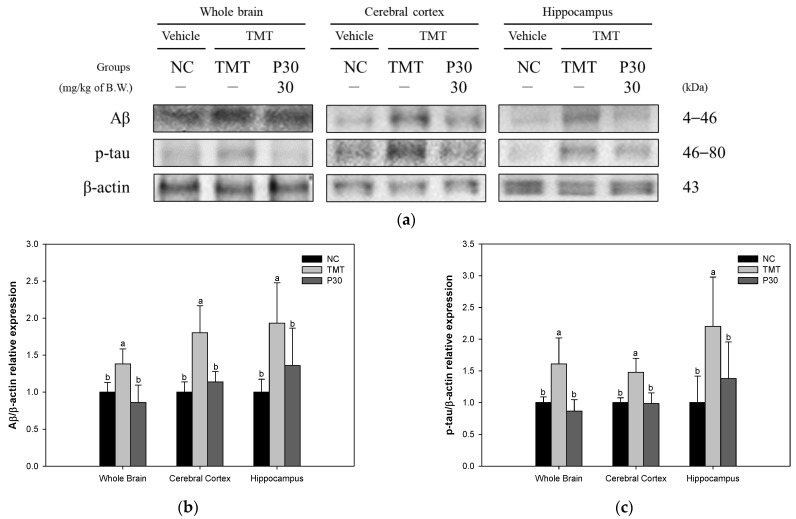
Effect of KRPBE on Aβ accumulation and hyperphosphorylation of tau in TMT-induced cognitive impaired mice. (**a**) Western blot band image: protein expression levels of (**b**) Aβ and (**c**) p-tau in the whole brain, cerebral cortex, and hippocampus. The results shown are mean ± SD (*n* = 3). Data were statistically considered at *p* < 0.05 and different small letters represent statistical differences.

The expression levels of Aβ accumulation and hyperphosphorylation of tau toxicity-induced apoptosis-related proteins were investigated using Western blot analysis to examine the effect of KRPBE (Figure 8). The Aβ expression level of the TMT group (138.12%, 180.35%, and 193.24%) was significantly increased compared to the NC group (Figure 8b). On the other hand, the P30 group (86.01%, 113.84%, and 135.92%) was significantly downregulated compared to the TMT group. As a result of examining the p-tau expression levels of the TMT group (160.83%, 147.61%, and 220.13%) were significantly increased compared to the NC group (Figure 8c). However, the P30 group (86.65%, 98.42%, and 137.93%) was significantly reduced compared to the TMT group.

### 2.6. Apoptosis-Relative Factors

**Figure 9:**
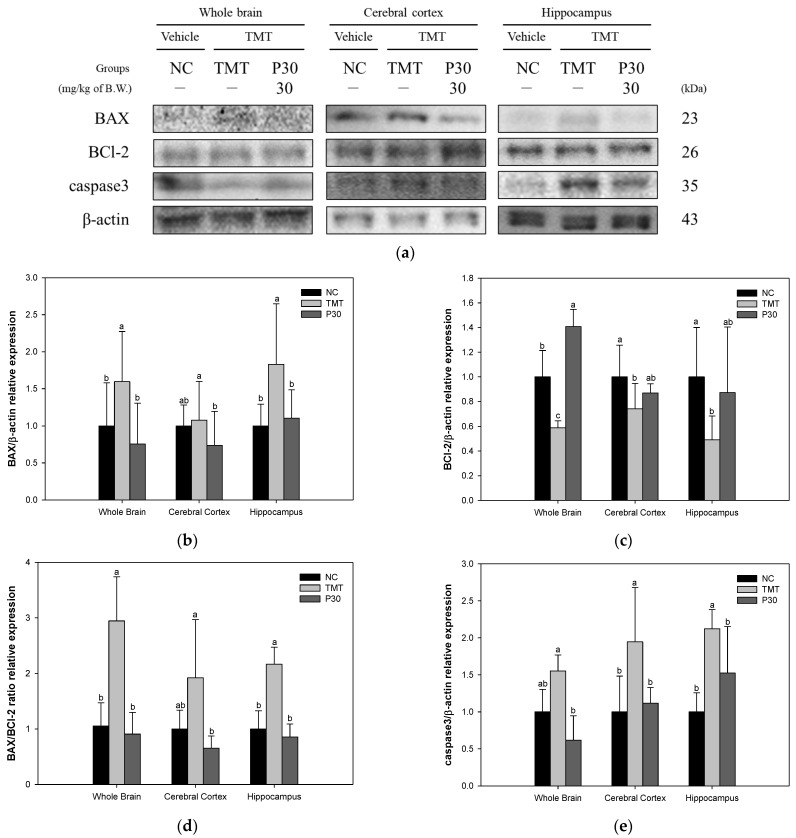
Effect of KRPBE on apoptosis in TMT-induced cognitive impaired mice. (**a**) Western blot band image: protein expression levels of (**b**) BAX; (**c**) BCl-2; (**d**) BAX/BCl-2 ratio; and (**e**) caspase 3 in whole brain, cerebral cortex, and hippocampus. The results shown are mean ± SD (*n* = 3). Data were statistically considered at *p* < 0.05 and different small letters represent statistical differences.

The expression level of apoptosis-related proteins was investigated using Western blot analysis to determine the neuroprotective effect of KRPBE (Figure 9). The BCl-2-associated X protein (BAX) expression level of the TMT group (159.49%, 107.46%, and 182.84%) was increased compared to the NC group (Figure 9b). On the other hand, the P30 group (75.48%, 73.73%, and 110.18%) showed a significant decrease compared to the TMT group. The expression level of B-cell lymphoma 2 (BCl-2) in the TMT group (58.88%, 74.18%, and 49.22%) was significantly decreased compared to the NC group (Figure 9c). However, the P30 group (140.76, 86.93%, and 87.30%) significantly improved compared to the TMT group. In addition, in the results expressed as a BAX/BCl-2 ratio, the TMT group (294.62%, 192.18%, and 216.51%) increased compared to the NC group (Figure 9d). On the other hand, the P30 group (90.77%, 65.19%, and 85.67%) showed a significant decrease compared to the TMT group. In addition, the expression level of caspase 3 of the TMT group (155.24%, 194.58%, and 212.25%) increased compared to the NC group (Figure 9e). The P30 group (61.74%, 111.70%, and 152.42%) presented a significant decrease compared to the TMT group.

### 2.7. Neuroinflammation-Relative Factors

**Figure 10:**
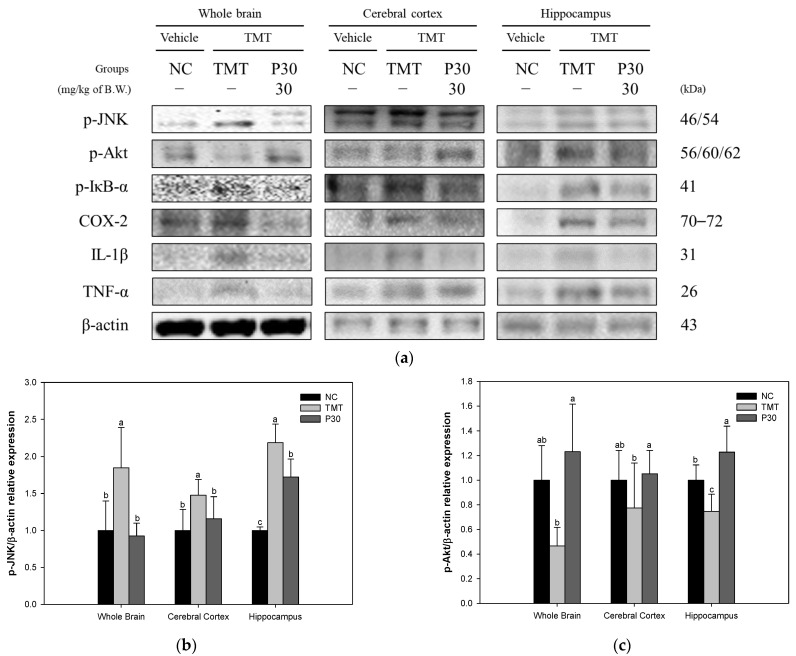

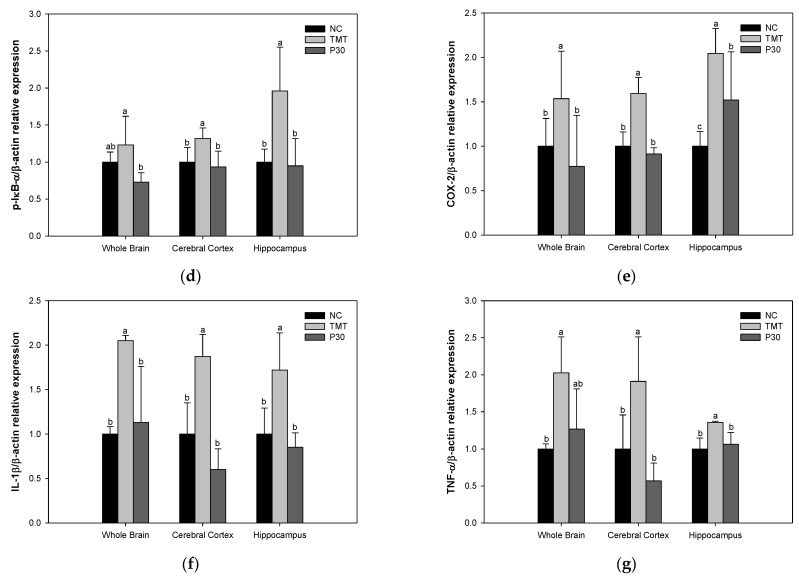
Effect of KRPBE on neuroinflammation in TMT-induced cognitive impaired mice. (**a**) Western blot band image: protein expression levels of (**b**) p-JNK; (**c**) p-Akt; (**d**) p-IκB-α; (**e**) COX-2; (**f**) IL-1β; and (**g**) TNF-α in the whole brain, cerebral cortex, and hippocampus. The results shown are mean ± SD (*n* = 3). Data were statistically considered at *p* < 0.05 and different small letters represent statistical differences.

The expression levels of inflammation-related protein were investigated using Western blot analysis (Figure 10). The phospho-c-Jun N-terminal kinase (p-JNK) expression levels of the TMT group (184.71%, 147.78%, and 218.79%) were significantly increased compared to the NC group (Figure 10b). However, the expression levels of p-JNK in the P30 group (92.52%, 115.88%, and 173.32%) were significantly reduced compared to the TMT group. On the other hand, the phospho-protein kinase B (p-Akt) expression level was decreased in all tissues of the TMT group (46.64%, 77.48%, and 74.74%) compared to the NC group (Figure 10c). The P30 group (123.12%, 105.17%, and 122.77%) was up-regulated compared to the TMT group. In addition, the phospho-nuclear factor of kappa light polypeptide gene enhancer in B-cells inhibitor-alpha (p-IκB-α), cyclooxygenase-2 (COX-2), and interleukin 1 beta (IL-1β) expression levels of the TMT group (123.14%, 131.76%, and 196.14%, p-IκB-α; 153.38%, 159.39%, and 204.55%, COX-2; and 204.99%, 187.16%, and 172.03%, IL-1β) were significantly increased compared to the NC group (Figure 10d–f). On the other hand, the expression levels of the P30 group (72.80%, 93.34%, and 94.84%, p-IκB-α; 77.44%, 91.30%, and 152.12%, COX-2; and 113.10%, 60.16%, and 85.24%; IL-1β) were significantly down-regulated. Also, the TNF-α expression level of the TMT group (202.61%, 191.04%, and 135.87%, respectively) was significantly increased compared to the NC group (Figure 10g). On the other hand, the P30 (126.92%, 56.89%, and 106.47%, respectively) was down-regulated compared to the TMT group.

## 3. Discussion

Alzheimer’s disease is the most common type of dementia and one of the most important medical and social problems among the elderly [1]. The major pathological features of AD include the deposition of Aβ, formation of neurofibrillary tangles, oxidative stress, mitochondrial dysfunction, and neuronal cell loss [4,18]. In particular, as direct evidence that oxidative stress increases in AD patients, there are studies confirming increased protein and DNA oxidation, increased lipid peroxidation, and decreased polyunsaturated fatty acids in AD brains [19]. Furthermore, the continuous attack of oxidative stress during the normal aging process is reported to cause an imbalance between ROS and antioxidant enzymes and is a major factor triggering the excessive production and deposition of Aβ in AD [20]. TMT is a substance that causes deficits in learning and memory formation by selectively causing neuronal damage in mouse brain regions, especially in the CA1 and CA3 regions of the hippocampus [13]. TMT is also used as a tool to derive a neuropathological model because it induces loss of the hippocampal CA3 region and inhibition of synaptic circuit connections, a pathological mechanism similar to neurodegenerative diseases such as AD [6]. Moreover, TMT-induced oxidative stress ultimately causes depletion of γ-aminobutyric acid (GABA) in synaptic vesicles and leads to neurotransmitter abnormalities [6]. In particular, TMT preferentially affects neurons in the hippocampus, pyriform cortex, and neocortex and neurodegeneration in these areas is accompanied by seizures, hyperactivity, and learning disabilities [21]. Therefore, in this study, the improvement mechanism of KRPBE against TMT-induced oxidative stress was investigated to confirm its utility as a functional food material.

Oxidative stress, one of the main pathological features of AD, induces Aβ deposition and hyperphosphorylation of tau and promotes various negative mechanisms such as ROS generation [4,22]. It is reported that the Aβ peptide, a major component of senile plaques induced by oxidative stress, activates an intracellular apoptosis pathway, leading to neuronal cell death [23]. The inflammatory response induced by microglia and astrocytes activated by ROS and Aβ promotes tau phosphorylation and polymerization, impairing neuron structure and function [24]. These senile plaques and NFTs and massive neuronal loss eventually lead to chronic and progressive memory impairment [23]. In addition, TMT-induced neurodegeneration is characterized by intensive oxidative stress and neuronal cell death in mouse brain regions, particularly in the hippocampus. Furthermore, TMT induced the degeneration of pyramidal neurons and caused neuronal loss in the pyriform cortex and entorhinal cortex, which are cortical regions connected to the hippocampus, resulting in increased memory impairment and learning deficits [6]. Therefore, the improvement effect of KRPBE on TMT-induced behavioral dysfunction was investigated in this study (Figure 1, Figure 2 and Figure 3). In the Y-maze, passive avoidance, and Morris water maze tests, treatment with KRPBE was shown to improve TMT-induced behavioral dysfunction by improving spatial task performance and short-term and long-term learning and memory. Similarly, the administration of phenol-rich pine bark extract improved spatial tasks and long-term and short-term memory abilities in scopolamine-induced rats [25]. Polyprenols from pine needles of *Pinus massoniana* improved memory and cognitive impairment by strengthening the oxidative defense system and regulating the production and dissimilation of Aβ-related enzymes [26]. In addition, in scopolamine induced amnesia mouse model, 30% ethanol extract of pine needle (*P. densiflora* Sieb et Zucc.) regulated cholinergic activity by promoting the expression levels of p-cAMP response element-binding protein (p-CREB) and brain-derived neurotrophic factor (BDNF), which ultimately showed an anti-amnestic effect [27]. PYC, standardized with procyanidins as its main ingredient, significantly improved memory deficit in the ischemia and reperfusion-induced memory deficit gerbil model by protecting CA1 pyramidal neurons with strong antioxidant activity [28]. Therefore, it is considered that KRPBE has a protective effect on TMT-induced cognitive decline in ICR mice, mainly by regulating antioxidant and cholinergic systems.

In the brains of AD patients, generated oxidative stress increases Aβ production and aggregation and promotes tau phosphorylation and polymerization, resulting in a vicious cycle that promotes various mechanisms including ROS production [4]. Also, an imbalance of the antioxidant defense system is caused due to oxidative stress such as ROS [10]. In particular, since brain membrane phospholipids are composed of polyunsaturated fatty acids, they are very vulnerable to free radicals and oxidative stress; oxidative stress increases lipid peroxidation [5]. Furthermore, oxidative damage or membrane deformation due to lipid peroxidation products alters the synaptic membrane and eventually leads to neuron death [13]. TMT also enhances ROS formation both in vivo and in vitro, leading to oxidative stress and mitochondrial damage in sensitive cells [29]. Therefore, the antioxidant system improvement effect of KRPBE on TMT-induced oxidative stress was investigated in this study (Figure 4). Treatment with KRPBE significantly increased the levels of SOD and reduced GSH, which are representative antioxidant biomarkers in the body, and reduced MDA content through the improvement of the antioxidant system. Moreover, *P. densiflora* needle ethanol extract was found to have antioxidant and anti-inflammatory effects by suppressing oxidative stress and the production of inflammatory mediators in lipopolysaccharide-induced RAW264.7 macrophages and in the arachidonic acid-induced ICR mouse model [30]. Pine needle extract significantly attenuated the products of lipid peroxidation in the hippocampus and serum of acute restraint stress mouse models and prevented memory impairment with the regulation of stress hormones, hippocampal excitotoxicity, and oxidative damage [31]. In addition, procyanidin B1, which contained the highest content among the phenolic compounds in KRPBE, had a neuroprotective effect by significantly reducing H_2_O_2_-induced ROS production and MDA levels in PC12 cells and Zebrafish. In addition, procyanidin B1, B2, and B3 identified in KRPBE significantly improved the antioxidant system by increasing catalase and SOD activities [32]. This suggests that the major bioactive substances contained in KRPBE have an ameliorating effect on TMT-induced oxidative stress through regulation of the antioxidant system.

The cholinergic system processes information in the cortex and hippocampus and the loss of synaptic plasticity involved in learning and memory affects cognitive function and behavior [33]. In particular, the activity of ChAT in the brains of AD patients was greatly reduced in the amygdala, hippocampus, and cortex and the concentration of acetylcholine at synapses was reduced [3]. This is because lipid peroxides, which are induced by ROS during neurodegeneration, increase AChE activity. In addition, ACh, which is directly affected by AChE activity, is a neurotransmitter present in the central nervous system and plays an important role in cognition and memory control. In other words, when ACh levels are lowered by excessive activity of AChE, it causes neuronal signal transmission disorders and memory loss [7]. There is a major correlation between the loss of these cholinergic neurons and synaptic loss [33]. In a recent study, oxidative stress was reported to be a major factor in synaptic dysfunction in the AD brain [34]. SYP and PSD-95 are key biomarkers of synaptic toxicity and they are reported to play important roles in synaptic development and plasticity by being located in the presynaptic and postsynaptic regions, respectively [33]. Furthermore, neuronal cell loss in AD is most prominent in the temporal and frontal cortices and these biomarkers are particularly markedly reduced in the temporal and frontal cortex and hippocampus [34]. Therefore, to investigate the improvement effect of KRPBE on TMT-induced cholinergic and synaptic dysfunction, the improvement effect of protein expression levels in forebrain tissue as well as cerebral cortex and hippocampus was investigated (Figure 5 and Figure 6). Treatment with KRPBE ameliorated the TMT-induced decrease in ACh content and excessive activity of AChE (Figure 5). In addition, the consumption of KRPBE significantly increased the expression levels of ChAT, SYP, and PSD-95 and decreased the expression level of AChE in cerebral cortex and hippocampal tissues (Figure 6). Similar to these results, needle essential oils from various *Pinus* species showed potential beneficial effects in the treatment of Alzheimer’s disease by significantly inhibiting AChE activity in vitro [35]. Additionally, protocatechuic acid, a major bioactive substance of KRPBE, had neuroprotective effects by improving AChE activity, antioxidant system, and inflammatory biomarkers in streptozotocin (STZ)-induced diabetic rats [36]. The administration of protocatechuic acid improved cognitive dysfunction by reducing IL-1β levels and increasing the expression of BDNF and SYP in the brains of SD rats induced by chronic intermittent hypoxia [37]. Taxifolin, another phenolic compound of KRPBE, ameliorated synaptic dysfunction by improving PSD-95 expression in an Aβ_1–42_-induced AD mouse model [38]. Therefore, KRPBE treatment for TMT-induced cognitive impairment could have a protective effect against cholinergic and synaptic toxicity caused by bioactive substances such as protocatechuic acid and taxifolin. It is considered that this ultimately helped restore signaling, learning, and memory functions.

Mitochondria regulate ATP production and intracellular Ca^2+^ homeostasis and play an important role in regulating ROS generation and apoptosis pathways [23]. Meanwhile, mitochondria are particularly vulnerable to oxidative stress and mitochondrial dysfunction is considered an important factor involved in the pathogenesis of AD [4]. Mitochondrial toxicity induced by oxidative stress and Aβ toxicity is reported to increase mitochondrial membrane permeability and disrupt calcium homeostasis [39]. Due to such mitochondrial dysfunction, reduced mitochondrial energy production and a chronic increase in oxidative stress ultimately initiate apoptosis [23]. Also, mitochondria concentrated at synaptic terminals, which are regarded as the starting point of the neurodegenerative process in Alzheimer’s disease, cause dysfunction by oxidative stress and Aβ, thereby activating the extrinsic apoptosis pathway [40]. Mitochondrial dysfunction also contributes to synaptic dysfunction and neurodegeneration [40]. Therefore, the improvement mechanism of KRPBE for mitochondrial dysfunction induced by TMT was investigated in this study (Figure 7). Similarly, vanillin, a major bioactive substance of KRPBE, had a neuroprotective effect by regulating intracellular ROS and MMP levels against rotenone-induced neurotoxicity in SH-SY5Y cells [41]. Taxifolin mitigated rotenone-induced neurotoxicity by attenuating mitochondrial complex 1 activity and the dysfunction of Na^+^/K^+^ATPase as well as by modulating neurotransmitter metabolism [42]. In addition, protocatechuic acid ameliorated mitochondrial dysfunction by improving mitochondrial membrane potential loss, ROS formation, and total GSH depletion in rotenone-induced PC12 cells [43]. In view of these results, it is considered that KRPBE has a neuroprotective effect on TMT-induced mitochondrial dysfunction.

In the Aβ precursor protein, oxidative stress promotes the expression and activity of beta and gamma secretases, which are important enzymes for Aβ production. Also, Aβ aggregates can be mediators that induce tau hyperphosphorylation and increased fatty acid oxidation in the brains of AD patients promotes tau polymerization [4]. The microtubule-associated protein tau is hyperphosphorylated three- to four-fold in AD, making it unsuitable for microtubule assembly as it detaches from microtubules and aggregates into NFTs [5]. In addition, synaptic dysfunction and the development of NFT due to neuronal cell death in the hippocampus and entorhinal cortex appear as major symptoms of AD. In particular, the accumulation of Aβ plaques and NFTs in the hippocampus and cortex in AD causes cognitive decline such as memory loss [44]. Therefore, in this study, the expression levels of Aβ and p-tau protein by TMT-induced oxidative stress were measured in the forebrain, cerebral cortex, and hippocampus (Figure 8). Treatment with KRPBE improves Aβ accumulation and tau hyperphosphorylation against TMT-induced toxicity in all tissues and it is considered that this can help improve the neurotoxicity mechanism induced by it. Similar to these results, taxifolin, a bioactive substance of KRPBE, alleviated oxidative tissue damage by suppressing the expression of Aβ and reducing the levels of pro-inflammatory cytokines in the brain of cerebral amyloid angiopathy (CAA) model mice [45]. Protocatechuic acid prevented PC12 cell death by inhibiting aggregation and fibril destabilization from Aβ and α-synuclein-induced toxicity [46]. In addition, treatment with caffeic acid helped to improve memory ability by maintaining synaptic activity by regulating glycogen synthase kinase-3 beta (GSK-3β) and tau protein expression and Aβ levels in the hippocampus of high-fat diet-induced hyperinsulinemia rats [47]. In particular, procyanidin 1 and procyanidins, the main bioactive substances of KRPBE, inhibited Aβ fibrillogenesis and significantly suppressed Aβ oligomer-induced neuronal cell death [48]. Therefore, the various bioactive substances contained in KRPBE treatment showed a neuroprotective effect by significantly improving oxidative stress-induced Aβ accumulation and hyperphosphorylated tau expression level. Its neuroprotective effects, particularly in the hippocampus and cortex, are suggested to have helped improve cognitive function.

Apoptosis is considered an important contributor to the pathology of neurodegeneration in AD [23]. Recent studies have reported that ROS and the resulting oxidative stress play a central role in apoptosis. In particular, oxidative stress resulting from an imbalance of biochemical defense mechanisms that neutralize ROS-mediated effects induces mitochondrial dysfunction and is considered one of the important mediators of apoptosis [49]. Oxidative stress-induced mitochondrial damage and Aβ accumulation upsets the balance of anti-apoptotic protection by down-regulating the anti-apoptotic factor BCl-2 and up-regulating BAX [50]. Precipitates of the accumulation of Aβ and activated caspase 3 were found in the brain tissues of AD patients. Also, other members of the caspase family caused apoptosis due to Aβ cytotoxicity, leading to neuronal loss in AD pathology [51]. Furthermore, TMT neurotoxicity induces oxidative stress and apoptosis, especially in hippocampal neurons, leading to hippocampal neurodegeneration [52]. Therefore, the expression levels of apoptosis factors in response to TMT-induced oxidative stress and Aβ accumulation were investigated in this study (Figure 9). Regarding TMT-induced apoptosis, KRPBE treatment was found to have an anti-apoptotic effect by regulating the BAX/BCl-2 ratio and caspase 3 expression level in all tissues. Procyanidin B2 detected in KRPBE inhibited lipopolysaccharide-induced apoptosis by inhibiting the BCl-2/BAX and nuclear factor kappa-light-chain-enhancer of activated B cells (NF-κB) signaling pathways in human umbilical vein endothelial cells [53]. In addition, vanillin was shown to exhibit neuroprotective effects by regulating inflammatory cytokines and BAX/BCl-2 in rotenone-induced SH-SY5Y neuroblastoma cells [54]. PYC, which has a bioactive substance similar to KRPBE, improved oxygen–glucose deprivation/reoxygenation (OGD/R)-induced apoptosis in primary rat astrocytes by regulating caspase 3 and BCl-2 expression [55]. In view of these results, the treatment with KRPBE showed a neuroprotective effect by having an anti-apoptotic effect on hippocampal and cortex neurons, which are particularly important for memory and learning ability.

Misfolded and aggregated proteins, such as oxidative stress-induced senile plaques and NFTs, bind to pattern recognition receptors on microglia and astroglia, leading to the release of inflammatory mediators involved in disease progression [56]. In the resulting neuroinflammation condition, microglia-driven IL-1β and TNF-α are activated [57]. Furthermore, oxidative stress and the accumulation of Aβ and tau increase inflammation and cytokine production, providing a mechanism to initiate and maintain a chronic inflammatory response [6]. Excessive release of pro-inflammatory mediators leads to neurodegeneration through various signaling pathways, including nuclear factor κB (NF-kB) and mitogen-activated protein kinase (MAPK) [58]. MAPKs, including JNK and p38, act as key biosynthetic regulators of pro-inflammatory cytokines such as COX-2, tumor necrosis factor-α, and IL-1β [58]. In addition, JNK, which is activated by pro-inflammatory cytokines and reactive oxygen species, exacerbates neuronal death signals and neurodegeneration [13,59]. p-JNK negatively affects downstream processes related to cell survival, such as the phosphoinositide 3-kinases (PI3K)/Akt cascade [60]. Inhibition of AKT activation results in NF-κB release and activated NF-kB can induce the transcription of COX-2 and inducible nitric oxide synthase (iNOS) [61,62]. Furthermore, activation of NF-κB results in the release of the inflammatory cytokines TNF-α and IL-1β [61]. Specifically, in the AD brain, COX-2 induces neurotoxicity by increasing the secretion of inflammatory cytokines in the cortex and hippocampus [63]. Therefore, the protective effect of KRPBE against TMT-induced neuroinflammation was investigated in this study (Figure 10). It was confirmed that the expression levels of neuroinflammatory factors in response to TMT-induced tau hyperphosphorylation were improved in all brain tissues due to KRPBE treatment. Similar to these results, *P. densiflora* needle extract had an anti-inflammatory effect by down-regulating inflammatory mediators such as IL-1β, TNF-α, COX-2, and PGE 2 in LPS-induced RAW 264.7 macrophages and arachidonic acid-induced ICR mouse model [30]. Furthermore, the pine (*Pinus morrisonicola* Hay.) needle contributed to the anti-inflammatory effect by inhibiting the generation of ROS and the expression levels of iNOS and COX-2 proteins in LPS-induced RAW 264.7 cells [64]. *P*. *densiflora* needle supercritical fluid extract helped anti-inflammatory activity by inhibiting the expression of iNOS, IL-6, and IL-1β, which are LPS-induced pro-inflammatory mediators, in RAW 264.7 macrophages [65]. Therefore, it is suggested that the treatment with KRPBE might contribute to the improvement of cognitive function by protecting neurons by regulating the excessive inflammatory response induced by TMT.

In summary, the anti-amnesic effect of KRPBE was investigated in mice TMT-induced cognitive impairment. KRPBE treatment significantly improved spatial workability, short-term and long-term learning, and memory abilities in behavioral tasks. In particular, it has a neuroprotective effect by remarkably improving antioxidant and cholinergic system imbalance and synaptic toxicity. In addition, it is thought to have helped improve cognitive dysfunction by improving mitochondrial dysfunction, apoptosis pathway, and neuroinflammation. Based on these results, it is expected to be used as an eco-friendly high value-added food with anti-amnesia effects. However, since studies related to synaptic plasticity are insufficient in this paper, it is necessary to evaluate research on synaptic plasticity by measuring biomarkers such as the CREB/BDNF pathway in the future. In addition, it is judged that further histological studies should be conducted.

## 4. Materials and Methods

### 4.1. Sample Preparation

KRPBE powder used in the experiment was provided by Nutrapharm Co., Ltd. (Yongin, Republic of Korea), on 11 May 2021. For the manufacture of KRPBE, the bark of *Pinus densiflora* is mixed with purified water using an extraction system and extracted. Then, it undergoes a concentration and sterilization process. The concentrated extract was powdered using a large spray dryer. In addition, according to a previous study, procyanidin B1, protocatechuic acid, catechin, taxifolin, vanillin, and caffeic acid were identified as major phenolic compounds of KRPBE by high-performance liquid chromatography (HPLC) analysis [15]. *Angelica gigas* Nakai root extract powder was purchased from Nutragen Inc. (Anyang, Republic of Korea) in January 2021, a commercial functional food for cognitive function, and used in the experiment.

### 4.2. Animal Experimental

#### Animal Experimental Design

ICR mice (male, 4 weeks old) were obtained from Samtako Inc. (Osan, Republic of Korea). The mice were reared in a constant environment (conditions: temperature of 22 ± 2 °C with relative humidity of 50–55%, day and night alternated at 12 h intervals). Mice (in vivo and ex vivo test, *n* = 7; mitochondrial function test, *n* = 5; Western blot, *n* = 3; total, *n* = 15) were randomly divided into 5 groups and the experiment was conducted. The NC and TMT groups were treated with the same volume of drinking water as the KRPBE-treated groups. The KRPBE groups were divided into low-concentration (15 mg/kg of body weight (B.W.), P15), high-concentration (30 mg/kg of B.W., P30), and normal sample (30 mg/kg of B.W., NS) groups for oral administration. KRPBE was treated by dissolving it in drinking water before the experiment. The PC group was treated by dissolving *Angelica gigas* Nakai root extract (30 mg/kg of B.W.) in drinking water for oral administration. Oral administration of all groups was performed once a day for 3 weeks. After 3 weeks of drinking water or KRPBE administration, TMT (7.1 μg/kg of B.W., 100 μL) dissolved in 0.89% NaCl solution was intraperitoneally injected to induce cognitive decline into TMT, P15, and P30 groups. In addition, the same volume of 0.85% NaCl was intraperitoneally injected into the NC and NS groups [66]. After the behavioral experiments, the mice were sacrificed using a CO_2_ inhalation device for ex vivo tests.

### 4.3. Animal Experimental

#### 4.3.1. Y-Maze Test

After 3 days for recovery, the Y-maze test was performed using a Y-shaped maze made of black plastic with three arms (length = 33 cm; height = 15 cm; and width = 10 cm). Each arm was randomly assigned to zones A, B, and C. The experiment was conducted after mice were placed at the end of designated one arm. The movement patterns and crossing behaviors of mice entering each arm were recorded with a video tracking system (Smart 3.0, Panlab, Barcelona, Spain) for 8 min [67].

#### 4.3.2. Passive Avoidance Test

The passive avoidance test conducted an experiment using two chambers divided into a light zone and a dark zone. The bottom of the chamber was made of stainless steel so that electric shocks could be applied and there was a door between the two chambers. During the training session, mice were placed in a bright chamber, turned off, and allowed to adapt to a dark environment for 1 min. Then, the light was turned on and the mice were exposed to bright light for 2 min. When the door between the two chambers was opened and the mouse moved from a bright zone to a dark zone on all 4 feet, an electric shock of 1.5 mA was given for 3 s. After 24 h of training session, the test was conducted in the same way as in the training session and the step-through latency of all 4 feet entering the dark chamber was measured for up to 300 s [68].

#### 4.3.3. Morris Water Maze Test

The Morris water maze test was conducted in a circular water pool with a diameter of 150 cm and a height of 60 cm. The circular water pool was filled with 30 cm of opaque water (23 ± 2 °C) mixed with squid ink (Cebesa, Valencia, Spain). In addition, it was divided into 4 parts as N, S, E, and W zones and visual clues were displayed on the walls of the tank along the divided quadrants. A black escape platform was placed in the center of the W zone and the location of the platform was not changed during the experiment. In the visible session, the platform was located 1 cm above the water surface and each mouse was allowed to swim for up to 60 s until it found the platform in each session. After that, in the hidden trial, the platform was submerged 1 cm below the water and the trial was performed for up to 60 s until the mouse found the platform. In the probe test, the platform was removed and the time spent of each mouse in the W zone was recorded using a video tracking system (Smart v3.0, Panlab SL, Barcelona, Spain) [69].

### 4.4. Preparation of Tissue

After the behavioral tests, brain tissue was immediately isolated. Brain tissue was homogenized with a bullet mixer (Next Advance Inc., Raymertown, NY, USA) with a 10-fold volume of phosphate buffered saline (PBS, pH 7.4). In addition, the brain tissue homogenate for reduced GSH measurement was homogenized by adding 10 mM phosphate buffer with 1 mM ethylenediaminetetraacetic acid (EDTA) corresponding to a 10-fold volume of the amount of the brain tissue. The protein concentration of homogenized samples was calculated according to the Bradford method [70].

### 4.5. Measurement of Antioxidant System Biomarker

To measure the SOD activity, the brain tissue homogenate was centrifuged 400× *g* for 10 min at 4 °C and the obtained pellet was mixed with 1× cell extraction buffer containing 20% (*v*/*v*) Triton X, distilled water, and 200 mM phenylmethylsulfonyl fluoride (pMSF). The mixture was placed on ice for 30 min and the mixture was vortexed every 5 min. Thereafter, the supernatant was obtained by centrifugation at 10,000× *g* at 4 °C for 10 min. The SOD content of the supernatant was measured according to the commercial SOD kit (Dojindo, Kumamoto, Japan).

To measure the content of reduced GSH, the homogenate was centrifuged at 10,000× *g* for 15 min at 4 °C to obtain a supernatant. The obtained supernatant and 5% metaphosphoric acid were mixed with a ratio of 1:1 and centrifuged at 2000× *g* for 2 min at 4 °C. Finally, the level of reduced GSH content was measured using the obtained supernatant. The supernatant was reacted with 0.26 M Tris-HCl (pH 7.5), 0.65 M NaOH, and 2 mg/mL OPT in MeOH for 15 min. The fluorescence of the reaction was measured at an excitation wavelength of 360 nm and an emission wavelength of 430 nm using a fluorescence photometer (Infinite F200, Tecan, Mannedorf, Switzerland) [66].

To measure the content of MDA, the brain homogenate was centrifuged at 2456× *g* for 10 min at 4 °C and the obtained supernatant was mixed with 1% phosphoric acid and 0.67% thiobarbituric acid (TBA) and reacted at 95 °C for 1 h. The mixture was centrifuged at 2356× *g* for 1 min at 4 °C. In addition, the supernatant was measured at 532 nm using a spectrophotometer (UV-1800, Shimadzu, Kyoto, Japan) [6].

### 4.6. Measurement of ACh Content and AChE Inhibitory Activity

To evaluate the ACh content, the brain homogenate was centrifuged at 13,572× *g* for 30 min at 4 °C and the obtained supernatant was reacted with an alkaline hydroxylamine reagent [2 M hydroxylamine in 1 M HCl and 3.5 M sodium hydroxide, (1:1 mixture)]. The mixture was then mixed with 0.5 M HCl and 0.37 M FeCl_3_·6H_2_O in 0.1 M HCl. The ACh content was measured at 540 nm using a microplate reader (Epoch2, BioTek Instrument Inc., Winooski, VT, USA) [71].

To evaluate the AChE activity, the brain homogenate was centrifuged at 13,772× *g* for 30 min at 4 °C and the obtained supernatant was reacted with an alkaline hydroxylamine reagent. In total, 50 mM sodium phosphate buffer (pH 8.0) was added to the previously centrifuged supernatant and incubated at 37 °C for 10 min by adding Ellman’s reaction mixture [500 μM acetylthiocholine with 1 mM 5,5-dithiobis-(2-nitrobenzoic acid) (DTNB)]. The reaction solution was measured at 405 nm for 20 min using a microplate reader (Epoch2, BioTek) [72].

### 4.7. Extraction of Mitochondria from Brain Tissure

To extract mitochondria, the brain was homogenized by adding mitochondrial isolation buffer (MI) [215 mM mannitol, 75 mM sucrose, 0.1% Bovine Serum Albumin (BSA), and 20 mM Hydroxyethyl piperazine Ethane Sulfonicacid (HEPES) sodium salt (pH 7.2)] and 1 mM ethylene glycol-bis(β-aminoethyl ether)-*N*,*N*,*N*′,*N*′-tetraacetic acid (EGTA). The obtained homogenate was centrifuged at 1300× *g* for 5 min at 4 °C to obtain a supernatant. The mixture was re-centrifuged at 13,000× *g* for 10 min at 4 °C and the pellet was mixed with 0.1% digitonin and 1 mM EGTA in MI buffer and reacted for 5 min. The reaction solution was centrifuged at 13,000× *g* for 15 min at 4 °C. Then, the pellet was mixed with MI buffer and centrifuged at 10,000× *g* for 10 min at 4 °C. Afterwards, the pellet was mixed with MI buffer again and the mixture was used for mitochondrial function evaluation [6].

### 4.8. Evaluation of Mitochondrial Activity

To evaluate the ROS level, mitochondrial extracts were reacted with respiration buffer (including 123 mM KCl, 2 mM KH_2_PO_4_, 2.5 mM malate, 20 mM HEPES, 1 mM MgCl_2_, 5 mM pyruvate, and 500 μM EGTA) and 25 μM 2′,7′-dichlorofluorescin diacetate (DCF-DA). Then, the reactants were incubated in the dark for 20 min at 4 °C and fluorescence was measured at 485 nm (excitation wavelength) and 535 nm (emission wavelength) using a fluorescence microplate reader (Infinite F200, Tecan) [66].

To measure the MMP level, the mitochondrial extract was reacted with MI buffer with 5 mM pyruvate, 5 mM malate, and 1 μM JC-1. The reaction was gently mixed and incubated in the dark for 20 min at 4 °C. Then, fluorescence was measured at 530 nm (excitation wavelength) and 590 nm (emission wavelength) using a fluorescence microplate reader (Infinite F200, Tecan) [13].

To measure the ATP level, the mitochondrial extract was evaluated using a commercial ATP kit (Sigma-Aldrich chemical Co., Milwaukee, WI, USA). The ATP level was assessed according to the manufacturer’s protocol and reactants were measured using a luminometer (Glomax^®^, Promega, Sunnyvale, CA, USA).

### 4.9. Western Blot

To measure the expression levels of proteins, the whole brain and sectioned hippocampus and cerebral cortex tissues were homogenized by mixing with Protein^Ex^ animal cell/tissue buffer (Gene All Biotechnology, Seoul, Republic of Korea) containing 1% protease inhibitor cocktail (Quartett, Berlin, Germany) and centrifuged at 13,000× *g* for 10 min at 4 °C. Afterwards, the supernatant was mixed with sample loading buffer (#1610374, Bio-Rad) and used for immunoblotting analysis. Samples were separated by sodium dodecyl sulfate-polyacrylamide gel electrophoresis (SDS-PAGE) and proteins separated according to size were transferred to a PVDF membrane (Millipore, Billerica, MA, USA). After that, it was treated with 5% skim milk for 1 h to block the membrane and then washed three times for 10 min each using tris-buffered saline (TBS) with 0.1% of Tween 20 (TBST buffer). The membrane and primary antibody (1:1000) were incubated at 4 °C for 12 h. Then, the secondary antibody (1:2500) was reacted with the membrane for 1 h at room temperature, washed three times for 10 min with TBTS buffer, and the luminescent band was chemically analyzed using an image analyzer (iBright CL1500, Invitrogen, Waltham, MA, USA). Also, antibody information used in this study is shown in Table 1 below.

### 4.10. Statistical Analysis

All data were expressed as mean ± SD and a one-way analysis of variance (ANOVA) was performed to calculate and analyze significant differences between groups and display them. Significant differences were confirmed by Duncan’s new multiple range test (*p* < 0.05) method through the SAS program (version 9.4, SAS Institute Inc., Cary, NC, USA). Other lowercase letters were then marked to indicate statistically significant differences.

## 5. Conclusions

**Figure 11:**
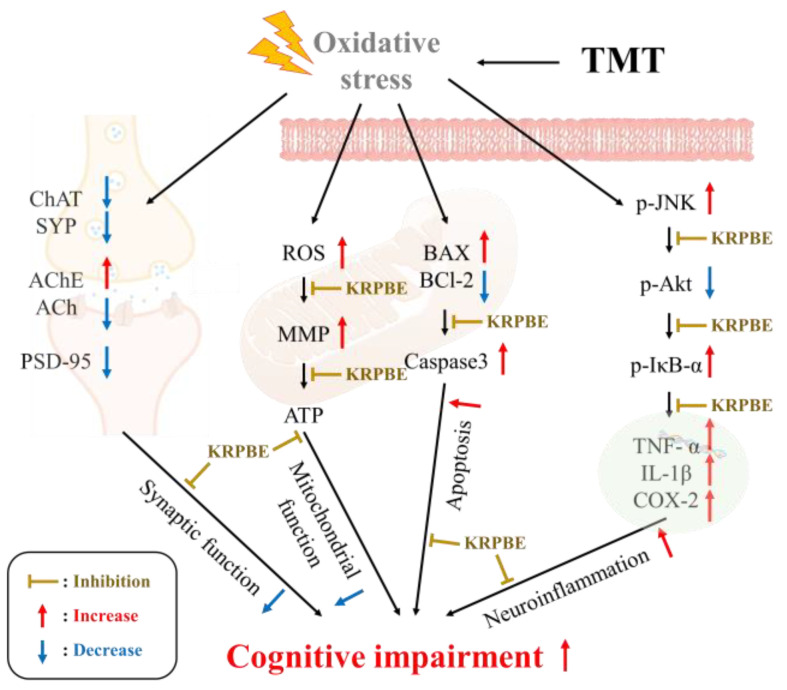
Improvement mechanism of KRPBE in TMT-induced cognitive impairment mice.

These results showed that the treatment with KRPBE improved the imbalance of the antioxidant system in mice with cognitive impairment induced by TMT. In particular, attenuation of cholinergic dysfunction and synaptic toxicity may have significantly helped to improve cognitive function. In addition, by restoring mitochondrial activity and regulating apoptosis and inflammatory responses, it has a neuroprotective effect, thereby significantly improving spatial work and long-term memory abilities (Figure 11). In view of these results, it was confirmed that pine bark, which is mostly discarded in forestry, can be used as high value-added functional food material having an anti-amnesia effect, as well as the possibility of development as an economical and environmental material. Finally, this study will be used as data to secure efficacy and functionality data in future human application tests.

## Figures and Tables

**Table 1:** List of antibody information used in this study.

Antibody	Catalog No.	Concentration	Manufacturer
β-actin	sc-69879	1:1000	Santa Cruz Biotechnology (Santa Cruz, CA, USA)
AChE	sc-373901	1:1000	Santa Cruz Biotechnology (Santa Cruz, CA, USA)
PSD-95	sc-32290	1:1000	Santa Cruz Biotechnology (Santa Cruz, CA, USA)
SYP	sc-17750	1:1000	Santa Cruz Biotechnology (Santa Cruz, CA, USA)
Aβ	sc-374527	1:1000	Santa Cruz Biotechnology (Santa Cruz, CA, USA)
p-Akt 1/2/3	sc-393887	1:1000	Santa Cruz Biotechnology (Santa Cruz, CA, USA)
p-JNK	sc-6254	1:1000	Santa Cruz Biotechnology (Santa Cruz, CA, USA)
p-IκB-α	sc-8404	1:1000	Santa Cruz Biotechnology (Santa Cruz, CA, USA)
COX-2	sc-376861	1:1000	Santa Cruz Biotechnology (Santa Cruz, CA, USA)
IL-1β	sc-373800	1:1000	Santa Cruz Biotechnology (Santa Cruz, CA, USA)
BAX	sc-7480	1:1000	Santa Cruz Biotechnology (Santa Cruz, CA, USA)
BCl-2	sc-509	1:1000	Santa Cruz Biotechnology (Santa Cruz, CA, USA)
caspase 3	sc-56053	1:1000	Santa Cruz Biotechnology (Santa Cruz, CA, USA)
ChAT	20747-1AP	1:1000	Proteintech Group (Rosemont, IL, USA)
Anti-rabbit	7074S	1:5000	Cell Signaling Technology (Beverly, MA, USA)
Anti-mouse	#1706516	1:5000	Bio-Rad (Richmond, CA, USA)

## Data Availability

The data presented in this study are available on request from the corresponding author.
